# Supplementary material for: Prevalence and Risk Factors of Elevated Blood Pressure and Elevated Blood Glucose among Residents of Kajiado County, Kenya: A Population-Based Cross-Sectional Survey
Source: Int J Environ Res Public Health. 2020 Sep 23;17(19):6957. doi: 10.3390/ijerph17196957 (PMC7579460; doi:10.3390/ijerph17196957)
Supplement: Supplementary file 1 [file ijerph-17-06957-s001.zip › suppl/ijerph-908445-supplementary final.docx]

**Table S1.** Comparison between participants with complete data and those with missing data based on background characteristics, Kajiado County, Kenya.

| **Participants with Complete Data** | | | | **Participants with Missing Data** | | | |
| --- | --- | --- | --- | --- | --- | --- | --- |
| **Variable** | **Unweighted**  ***n*** | **Weighted**  ***n*** | **Weighted**  **%** | **Unweighted**  ***n*** | **Weighted**  ***n*** | **Weighted**  **%** | ***p-*Value** |
| **Total** | 593 | 601 |  | 157 | 149 |  |  |
| Age (years) |  |  |  |  |  |  | 0.63 |
| 25–34 | 283 | 236 | 39.6 | 78 | 61 | 40.6 |  |
| 35–54 | 236 | 250 | 40.9 | 60 | 66 | 44.8 |  |
| 55–64 | 74 | 115 | 19.5 | 19 | 22 | 14.6 |  |
| Gender |  |  |  |  |  |  | 0.32 |
| Men | 221 | 319 | 53.1 | 47 | 72 | 48.4 |  |
| Women | 372 | 282 | 46.9 | 110 | 77 | 51.6 |  |
| Marital status |  |  |  |  |  |  | 0.41 |
| Single (Never married/  divorced/widowed) | 121 | 98 | 16.3 | 38 | 30 | 20.4 |  |
| Married | 472 | 503 | 83.7 | 119 | 119 | 79.6 |  |
| Ethnicity |  |  |  |  |  |  | 0.78 |
| Maasai | 200 | 259 | 43.1 | 53 | 67 | 44.7 |  |
| Kikuyu | 140 | 117 | 19.5 | 35 | 24 | 16.1 |  |
| Other | 253 | 225 | 37.4 | 69 | 58 | 39.2 |  |
| Residence |  |  |  |  |  |  | 0.88 |
| Rural | 303 | 373 | 62.1 | 72 | 91 | 60.9 |  |
| Urban | 290 | 228 | 37.9 | 85 | 58 | 39.1 |  |
| Education |  |  |  |  |  |  | 0.99 |
| None | 130 | 167 | 27.8 | 33 | 41 | 27.4 |  |
| primary | 223 | 213 | 35.5 | 58 | 53 | 35.6 |  |
| Secondary/higher | 240 | 221 | 36.7 | 66 | 55 | 37.0 |  |
| Occupation |  |  |  |  |  |  | 0.95 |
| Unemployed | 128 | 136 | 22.6 | 33 | 32 | 21.4 |  |
| Employed unskilled | 77 | 72 | 11.9 | 21 | 18 | 12.0 |  |
| Employed skilled | 69 | 70 | 11.7 | 20 | 16 | 10.7 |  |
| Self Employed | 319 | 323 | 53.8 | 83 | 83 | 55.9 |  |
| Smoking |  |  |  |  |  |  | 0.41 |
| Never | 520 | 506 | 84.2 | 142 | 131 | 88.8 |  |
| Past | 30 | 40 | 6.3 | 3 | 3 | 1.8 |  |
| Current | 43 | 55 | 9.1 | 10 | 14 | 9.0 |  |
| Missing data | 0 | 0 | 0 | 2 | 1 | 0.4 |  |
| Alcohol consumption |  |  |  |  |  |  | 0.31 |
| Never | 438 | 422 | 70.2 | 106 | 90 | 60.5 |  |
| Past | 85 | 86 | 14.3 | 24 | 25 | 17.3 |  |
| Current | 70 | 93 | 15.5 | 26 | 33 | 22.2 |  |
| Missing data | 0 | 0 | 0 | 1 | 1 | N/A |  |
| Adequate physical activity |  |  |  |  |  |  | 0.96 |
| Yes | 222 | 237 | 39.5 | 56 | 46 | 38.5 |  |
| No | 355 | 348 | 57.9 | 94 | 83 | 57.1 |  |
| Missing data | 16 | 16 | 2.6 | 7 | 20 | 4.4 |  |
| Fruits and vegetables daily intake |  |  |  |  |  |  | 0.24 |
| Yes | 105 | 92 | 15.3 | 34 | 31 | 20.9 |  |
| No | 482 | 505 | 83.1 | 119 | 115 | 76.9 |  |
| Missing data | 6 | 4 | 0.6 | 4 | 3 | 2.2 |  |
| High sugary foods/drinks use |  |  |  |  |  |  |  |
| Daily | 87 | 78 | 13.1 | 15 | 11 | 7.7 | 0.28 |
| Weekly | 221 | 218 | 36.2 | 57 | 47 | 31.8 |  |
| Occasionally | 277 | 297 | 49.4 | 82 | 88 | 58.7 |  |
| Missing data | 8 | 8 | 1.3 | 3 | 3 | 1.8 |  |
| Use cooking fat/oil |  |  |  |  |  |  | 0.92 |
| Mainly use cooking oil | 426 | 399 | 66.3 | 114 | 96 | 62.7 |  |
| Mainly use cooking fat | 153 | 189 | 31.5 | 39 | 47 | 27.5 |  |
| Missing data | 14 | 13 | 2.2 | 4 | 6 | 9.8 |  |
| Ever measured blood pressure |  |  |  |  |  |  | 0.42 |
| Yes | 307 | 310 | 49.8 | 86 | 75 | 50.5 |  |
| No | 277 | 281 | 45.9 | 68 | 72 | 48.0 |  |
| Missing data | 9 | 10 | 1.7 | 3 | 2 | 1.5 |  |
| Ever measured blood glucose |  |  |  |  |  |  | 0.93 |
| Yes | 180 | 230 | 38.2 | 53 | 59 | 39.4 |  |
| No | 405 | 364 | 60.6 | 100 | 88 | 59.0 |  |
| Missing data | 8 | 7 | 1.2 | 4 | 2 | 1.6 |  |

N/A: Percentage could not be computed due to very few participants with missing data.

**Table S2.** Bivariate analysis of risk factors of elevated blood pressure among the residents of Kajiado County, Kenya (*n* = 593).

| **Total (*n* = 593)** | | | **Men (*n* = 221)** | | **Women (*n* = 372)** | |
| --- | --- | --- | --- | --- | --- | --- |
| **Variable** | **OR (95% CI)** | ***p-*Value** | **OR (95% CI)** | ***p-*Value** | **OR (95% CI)** | ***p-*Value** |
| Age (years) |  |  |  |  |  |  |
| 25–34 | 1 (Ref.) |  | 1 (Ref.) |  | 1 (Ref.) |  |
| 35–54 | 1.45 (1.00–2.12) | 0.05 | 1.14 (0.57–2.30) | 0.71 | 1.61 (1.03–2.53) | 0.04 |
| 55–64 | 4.01 (1.92– 8.38) | <0.01 | 2.61 (0.84–8.12) | 0.10 | 4.52 (1.68–12.15) | <0.01 |
| Gender |  |  |  |  |  |  |
| Women | 1 (Ref.) |  |  |  |  |  |
| Men | 2.10 (1.42–3.11) | <0.01 | N/A | N/A | N/A | N/A |
| Ethnicity |  |  |  |  |  |  |
| Maasai | 1 (Ref.) |  | 1 (Ref.) |  | 1 (Ref.) |  |
| Kikuyu | 2.25 (1.38–3.66) | <0.01 | 2.00 (0.73–5.48) | 0.18 | 2.68 (1.52–4.74) | <0.01 |
| Other | 2.01 (1.35–3.00) | <0.01 | 1.38 (0.68–2.82) | 0.37 | 2.41 (1.46–3.97) | <0.01 |
| Residence |  |  |  |  |  |  |
| Rural | 1 (Ref.) |  | 1 (Ref.) |  | 1 (Ref.) |  |
| Urban | 1.67 (1.16–2.38) | 0.01 | 1.14 (0.59–2.20) | 0.69 | 1.98 (1.28–3.07) | <0.01 |
| Education |  |  |  |  |  |  |
| Basic | 1 (Ref.) |  | 1 (Ref.) |  | 1 (Ref.) |  |
| High school/higher | 0.81 (0.57–1.16) | 0.26 | 0.59 (0.31–1.15) | 0.12 | 0.86 (0.55–1.33) | 0.49 |
| Occupation |  |  |  |  |  |  |
| Unemployed | 1 (Ref.) |  | 1 (Ref.) |  | 1 (Ref.) |  |
| Employed | 1.49 (0.91–2.44) | 0.12 | 2.12 (0.76–5.87) | 0.15 | 0.91 (0.49–1.68) | 0.75 |
| Self Employed | 2.15 (1.39–3.33) | <0.01 | 2.83 (1.05–7.63) | 0.04 | 1.78 (1.09–2.92) | 0.02 |
| Smoking |  |  |  |  |  |  |
| Not Smoking | 1 (Ref.) |  | 1 (Ref.) |  | *** |  |
| Currently Smoking | 2.26 (0.99–5.19) | 0.05 | 1.12 (0.45–2.73) | 0.81 | *** |  |
| Alcohol consumption |  |  |  |  |  |  |
| Not drinking | 1 (Ref.) |  | 1 (Ref.) |  | 1 (Ref.) |  |
| Currently drinking | 1.97 (1.05–3.70) | 0.04 | 1.59 (0.69–3.66) | 0.28 | 1.30 (0.45–3.79) | 0.63 |
| Adequate physical activity |  |  |  |  |  |  |
| Yes | 1(Ref.) |  | 1(Ref.) |  | 1 (Ref.) |  |
| No | 1.02(0.71–1.47) | 0.91 | 1.05(0.54–2.04) | 0.89 | 1.16 (0.74–1.83) | 0.52 |
| Missing data | 0.93 (0.31–2.78) | 0.90 | 1.05 (0.11–9.90) | 0.97 | 1.04 (0.29–3.74) | 0.96 |
| Fruits and vegetables daily intake |  |  |  |  |  |  |
| Yes | 1 (Ref.) |  | 1 (Ref.) |  | 1 (Ref.) |  |
| No | 0.94 (0.59–1.50) | 0.80 | 1.31 (0.57– 3.02) | 0.52 | 0.79 (0.45–1.40) | 0.43 |
| Missing data | 1.60 (0.17–14.91) | 0.68 | 1 (empty) | * | 1.34 (0.13–13.65) | 0.81 |
| Use of high sugary foods/beverages |  |  |  |  |  |  |
| Daily | 1 (Ref.) |  | 1 (Ref.) |  | 1 (Ref.) |  |
| Weekly | 0.79 (0.45–1.38) | 0.41 | 1.14 (0.40–3.24) | 0.81 | 0.63 (0.33–1.22) | 0.17 |
| Occasionally | 0.88 (0.51–1.52) | 0.66 | 1.08 (0.39–2.99) | 0.89 | 0.78 (0.41–1.49) | 0.46 |
| Missing data | 0.60 (0.13–2.71) | 0.51 | 1 (empty) | * | 1.01 (0.18–5.73) | 0.99 |
| Use cooking fat/oil |  |  |  |  |  |  |
| Mainly use cooking oil | 1 (Ref.) |  | 1 (Ref.) |  | 1 (Ref.) |  |
| Mainly use cooking fat | 0.82 (0.55– 1.22) | 0.33 | 0.88 (0.43–1.77) | 0.72 | 0.68 (0.41–1.13) | 0.14 |
| Missing data | 1.09 (0.34–3.50) | 0.88 | 0.48 (0.08–2.75) | 0.41 | 1.74 (0.35–8.54) | 0.50 |
| Body mass index (kg/m^2^) |  |  |  |  |  |  |
| Normal (<25.0) | 1 (Ref.) |  | 1 (Ref.) |  | 1 (Ref.) |  |
| Overweight (25.0–29.9) | 1.61 (1.07–2.43) | 0.02 | 2.64 (1.10–6.37) | 0.03 | 1.72 (1.04–2.83) | 0.03 |
| Obese (30.0 and above) | 2.92 (1.77–4.83) | <0.01 | 12.33 (1.63– 93.55) | 0.02 | 3.21 (1.82–5.66) | <0.01 |
| Waist Hip ratio |  |  |  |  |  |  |
| Normal (<0.9/men and < 0.85/women) | 1 (Ref.) |  | 1 (Ref.) |  | 1 (Ref.) |  |
| High (≥0.9/men and ≥0.85/women) | 1.63 (1.14–2.34) | 0.01 | 2.09 (1.04–4.20) | 0.04 | 1.73 (1.12–2.67) | 0.01 |
| Missing data | 2.44 (0.52–11.52) | 0.26 | N/A | * | 1.46 (0.26–8.22) | 0.67 |
| HbA1c (%) |  |  |  |  |  |  |
| Normal (< 6.0) | 1 (Ref.) |  | 1 (Ref.) |  | 1(Ref.) |  |
| Prediabetes (6.0–6.4) | 1.29 (0.70–2.35) | 0.41 | 2.59 (0.58–11.61) | 0.21 | 1.13 (0.57–2.24) | 0.73 |
| Diabetes (≥ 6.5) | 3.89 (1.36–11.14) | 0.01 | 3.45 (0.44–27.32) | 0.24 | 4.29 (1.26–14.7) | 0.02 |
| Total Cholesterol (mg/dl) |  |  |  |  |  |  |
| Optimal | 1 (Ref.) |  | 1 (Ref.) |  | 1 (Ref.) |  |
| High (≥240) | 5.50 (1.29–23.49) | 0.02 | 3.51 (0.4–27.56) | 0.23 | 6.73 (0.87–52.35) | 0.07 |
| Low Density Lipoprotein (mg/dl) |  |  |  |  |  |  |
| Optimal (<100) | 1 (Ref.) |  | 1 (Ref.) |  | 1 (Ref.) |  |
| High (≥130) | 1.34 (0.76–2.36) | 0.31 | 1.60 (0.58–4.42) | 0.36 | 1.11 (0.55–2.25) | 0.77 |
| Missing data | 1.27 (0.58–2.77) | 0.55 | 1.66 (0.19–14.20) | 0.64 | 1.39 (0.59–3.26) | 0.45 |
| High Density Lipoprotein (mg/dl) |  |  |  |  |  |  |
| Optimal | 1 (Ref.) |  | 1 (Ref.) |  | 1 (Ref.) |  |
| High (>60) | 1.13 (0.63–2.05) | 0.68 | 1.23 (0.43–3.58) | 0.70 | 1.08 (0.53–2.24) | 0.83 |
| Missing data | 1.37 (0.53–3.51) | 0.51 | 1.87 (0.18–19.53) | 0.60 | 1.48 (0.51–4.30) | 0.47 |
| Sodium–potassium ratio |  |  |  |  |  |  |
| Lower (≤3.1) | 1 (Ref.) |  | 1 (Ref.) |  | 1 (Ref.) |  |
| Higher (≥3.2) | 1.15 (0.81–1.64) | 0.44 | 0.71 (0.37–1.39) | 0.32 | 1.32 (0.86– 2.03) | 0.21 |

CI: Confidence Interval; Ref: Reference group; The Percentages and whole numbers might not sum up to due to rounding off. N/A: Confidence interval could not be computed due to very few participants with missing data. * p-value could not be computed due to very few participants with missing data. ^***^ Smoking among women was not included in the model due to the small number of smokers among the women.

**Table S3.** Bivariate analysis of risk factors of elevated blood glucose among the residents of Kajiado County, Kenya (*n* = 593).

| **Variable** | **Total (*n* = 593)** | | **Men (*n* = 221)** | | **Women (*n* = 372)** | |
| --- | --- | --- | --- | --- | --- | --- |
|  | **OR (95% CI)** | ***p-*Value** | **OR (95% CI)** | ***p-*Value** | **OR (95% CI)** | ***p-*Value** |
| Age(years) |  |  |  |  |  |  |
| 25–34 | 1 (Ref.) |  | 1 (Ref.) |  | 1 (Ref.) |  |
| 35–54 | 1.95 (1.18–3.23) | 0.01 | 2.79 (1.08–7.20) | 0.03 | 1.69 (0.92–3.09) | 0.09 |
| 55–64 | 5.03 (2.74–9.26) | <0.01 | 5.29 (1.87–14.99) | <0.01 | 5.92 (2.68–13.11) | <0.01 |
| Gender |  |  |  |  |  |  |
| Women | 1 (Ref.) |  | N/A | N/A | N/A | N/A |
| Men | 0.81 (0.52–1.28) | 0.38 |  |  |  |  |
| Ethnicity |  |  |  |  |  |  |
| Maasai | 1 (Ref.) |  | 1 (Ref.) |  | 1 (Ref.) |  |
| Kikuyu | 0.91 (0.51–1.64) | 0.75 | 0.97 (0.35–2.65) | 0.95 | 0.89 (0.43–1.83) | 0.74 |
| Other | 1.00 (0.61–1.64) | 0.10 | 0.70 (0.30–1.61) | 0.40 | 1.23 (0.66–2.27) | 0.52 |
| Residence |  |  |  |  |  |  |
| Rural | 1 (Ref.) |  | 1 (Ref.) |  | 1 (Ref.) |  |
| Urban | 0.89 (0.58–1.37) | 0.60 | 0.54 (0.25–1.15) | 0.11 | 1.15 (0.67–1.96) | 0.61 |
| Education |  |  |  |  |  |  |
| Basic | 1 (Ref.) |  | 1 (Ref.) |  | 1 (Ref.) |  |
| High school/higher | 0.66 (0.42–1.04) | 0.07 | 0.87 (0.41–1.85) | 0.73 | 0.57 (0.31–1.02) | 0.06 |
| Occupation |  |  |  |  |  |  |
| Unemployed | 1 (Ref.) |  | 1 (Ref.) |  | 1 (Ref.) |  |
| Employed | 0.35 (0.17–0.69) | <0.01 | 0.28 (0.08–1.01) | 0.05 | 0.45 (0.19–1.06) | 0.07 |
| Self Employed | 0.68 (0 .41–1.12) | 0.13 | 0.77 (0.26–2.32) | 0.64 | 0.64 (0.36–1.15) | 0.13 |
| Smoking |  |  |  |  |  |  |
| Not Smoking | 1 (Ref.) |  | 1 (Ref.) |  | 1 (Ref.) |  |
| Currently Smoking | 1.15 (0.52–2.57) | 0.73 | 1.13 (0.43–2.95) | 0.81 | 2.36 (0.42–3.16) | 0.33 |
| Alcohol consumption |  |  |  |  |  |  |
| Not drinking | 1 (Ref.) |  | 1 (Ref.) |  | 1 (Ref.) |  |
| Currently drinking | 0.81 (0.40–1.65) | 0.57 | 0.52 (0.19–1.42) | 0.20 | 2.01 (0.68–5.90) | 0.21 |
| Adequate physical activity |  |  |  |  |  |  |
| Yes | 1 (Ref.) |  | 1 (Ref.) |  | 1 (Ref.) |  |
| No | 1.43 (0.90–2.29) | 0.13 | 1.34 (0.63–2.87) | 0.45 | 1.45 (0.79–2.66) | 0.23 |
| Missing data | 2.13 (0.65– 7.05) | 0.21 | 1.69 (0.18–16.34) | 0.65 | 2.29 (0.55–9.52) | 0.25 |
| Fruits and vegetables daily intake |  |  |  |  |  |  |
| Yes | 1 (Ref.) |  | 1 (Ref.) |  | 1 (Ref.) |  |
| No | 0.76 (0.44–1.30) | 0.31 | 1.16 (0.41–3.22) | 0.78 | 0.63 (0.34–1.20) | 0.16 |
| Missing data | 1.00 (0.11–9.42) | 1.00 | N/A | * | 1.08 (0.11–11.15) | 0.95 |
| Use of high sugary foods/beverages |  |  |  |  |  |  |
| Daily | 1 (Ref.) |  | 1 (Ref.) |  | 1 (Ref.) |  |
| Weekly | 1.55 (0.73–3.27) | 0.25 | 6.00 (0.76–47.46) | 0.09 | 1.04 (0.45–2.43) | 0.93 |
| Occasionally | 1.78 (0.86– 3.67) | 0.12 | 4.91 (0.62–38.74) | 0.13 | 1.46 (0.66–3.24) | 0.35 |
| Missing data | 1 (empty) | * | N/A | * | N/A | * |
| Use cooking fat/oil |  |  |  |  |  |  |
| Mainly use cooking oil | 1 (Ref.) |  | 1 (Ref.) |  | 1 (Ref.) |  |
| Mainly use cooking fat | 0.83 (0.50–1.37) | 0.46 | 1.22 (0.56–2.65) | 0.61 | 0.64 (0.32–1.28) | 0.21 |
| Missing data | 0.33 (0.04–2.57) | 0.29 | N/A | * | 0.52 (0.06–4.27) | 0.55 |
| Body mass index (Kg/m2) |  |  |  |  |  |  |
| Normal (<25.0) | 1 (Ref.) |  | 1 (Ref.) |  | 1 (Ref.) |  |
| Overweight (25.0–29.9) | 1.27 (0.71– 2.27) | 0.43 | 2.46 (0.98–6.20) | 0.06 | 0.82 (0.38–1.75) | 0.61 |
| Obese (30.0 and above) | 4.24 (2.52–7.14) | <0.01 | 6.34 (2.48–16.23) | <0.01 | 3.30 (1.75–6.25) | <0.01 |
| Waist Hip ratio |  |  |  |  |  |  |
| Normal (<0.9/men and < 0.85/women) | 1 (Ref.) |  | 1 (Ref.) |  | 1 (Ref.) |  |
| High (≥0.9/men and ≥0.85/women) | 4.20 (2.49–7.08) | <0.01 | 3.59 (1.56–8.21) | <0.01 | 4.55 (2.29–9.04) | <0.01 |
| Missing data | 2.81 (0.57–13.90) | 0.21 | 8.15 (1.20–55.23) | 0.03 | N/A | * |
| Blood Pressure (mmHg) |  |  |  |  |  |  |
| Normal (<120 and <80) | 1 (Ref.) |  | 1 (Ref.) |  | 1 (Ref.) |  |
| Pre-Hypertension  (120–139/80–89) | 1.30 (0.73–2.32) | 0.37 | 2.33 (0.65–8.44) | 0.20 | 1.12 (0.57–2.21) | 0.73 |
| Hypertension  (≥140 and ≥90) | 2.90 (1.61– 5.23) | <0.01 | 3.92 (1.05–14.56) | 0.04 | 3.03 (1.52–6.04) | <0.01 |
| Total Cholesterol (mg/dl) |  |  |  |  |  |  |
| Optimal | 1 (Ref.) |  | 1 (Ref.) |  | 1 (Ref.) |  |
| High (≥240) | 3.15 (1.40–7.11) | 0.01 | 3.55 (1.11–11.37) | 0.03 | 3.05 (0.97–9.65) | 0.06 |
| Low Density Lipoprotein (mg/dl) |  |  |  |  |  |  |
| Optimal (<100) | 1 (Ref.) |  | 1 (Ref.) |  | 1 (Ref.) |  |
| High (≥130) | 1.85 (1.03–3.32) | 0.04 | 1.27 (0.48–3.36) | 0.63 | 2.44 (1.16–5.15) | 0.02 |
| Missing data | 1.67 (0.73–3.82) | 0.22 | 0.99 (0.11–8.54) | 0.99 | 1.83 (0.74–4.55) | 0.19 |
| High Density Lipoprotein (mg/dl) |  |  |  |  |  |  |
| Optimal | 1 (Ref.) |  | 1 (Ref.) |  | 1 (Ref.) |  |
| High (>60) | 0.78 (0.38–1.56) | 0.48 | 0.52 (0.18–1.54) | 0.24 | 1.00 (0.40–2.52) | 1.00 |
| Missing data | 1.21 (0.43–3.39) | 0.71 | 0.53 (0.05–5.55) | 0.60 | 1.61 (0.47–5.49) | 0.45 |
| Sodium–potassium ratio |  |  |  |  |  |  |
| Lower (≤3.1) | 1 (Ref.) |  | 1 (Ref.) |  | 1 (Ref.) |  |
| Higher (≥3.2) | 0.81 (0.53–1.25) | 0.35 | 0.79 (0.38–1.66) | 0.54 | 0.84 (0.49–1.44) | 0.52 |

CI: Confidence Interval***.*** The Percentages and whole numbers might not sum up to due to rounding off. AOR: Adjusted Odds Ratio. N/A: Confidence interval could not be computed due to very few participants with missing data. * *p–*value could not be computed due to very few participants with missing data.  ^***^ Smoking among women was not included in the model due to the small number of smokers among the women.

**Table S4.** Summary of the distribution levels of elevated blood pressure and elevated blood glucose by age and body mass index among the residents of Kajiado county, Kenya (*n* = 593).

| **Variables** | **Elevated Blood Pressure** | | | | | | | **Elevated Blood Glucose** | | | | | |
| --- | --- | --- | --- | --- | --- | --- | --- | --- | --- | --- | --- | --- | --- |
|  | **Men** | | | **Women** | | | **Men** | | | | **Women** | | |
|  | **Normal**  ***n* = 45** | **Pre-**  **Hypertension**  ***n* = 112** | **Hypertension**  ***n* = 64** | **Normal**  ***n* = 130** | **Pre-**  **Hypertension**  ***n* = 159** | **Hypertension**  ***n* = 83** | **Normal**  ***n* = 188** | | **Pre-**  **Diabetes**  ***n* = 20** | **Diabetes**  ***n* = 13** | **Normal**  ***n* = 306** | **Pre-**  **Diabetes**  ***n* = 41** | **Diabetes**  ***n* = 25** |
|  | ***n* (%)** | ***n* (%)** | ***n* (%)** | ***n* (%)** | ***n* (%)** | ***n* (%)** | ***n* (%)** | | ***n* (%)** | ***n* (%)** | ***n* (%)** | ***n* (%)** | ***n* (%)** |
| Age (years) |  |  |  |  |  |  |  | |  |  |  |  |  |
| 25–34 | 23 (46.9) | 57 (39.2) | 18 (24.8) | 78(54.5) | 81(41.3) | 26 (28.9) | 91(40.2) | | 4 (15.1) | 3 (15.8) | 163(46.1) | 15 (34.0) | 7 (31.2) |
| 35–54 | 18 (44.7) | 40 (37.4) | 27(33.9) | 47(37.9) | 62 (45.7) | 42 (57.4) | 70 (36.3) | | 9 (40.3) | 6 (49.3) | 123 (45.7) | 19 (46.0) | 9 (38.9) |
| 55–64 | 4 (8.4) | 15 (23.4) | 19 (41.3) | 5(7.6) | 16 (13.0) | 15(13.7) | 27 (23.4) | | 7 (44.7) | 4 (34.9) | 20 (8.2) | 7 (20.0) | 9 (29.9) |
| BMI |  |  |  |  |  |  |  | |  |  |  |  |  |
| Underweight (<18.5) | 6 (19.1) | 8 (6.8) | 4 (9.5) | 9 (12.4) | 4 (2.9) | 3 (6.5) | 15 (10.9) | | 3 (11.4) | 0 (0.0) | 10 (4.6) | 4 (21.4) | 2 (4.8) |
| Normal (18.5–24.9) | 31 (59.6) | 61 (56.7) | 23 (34.4) | 57 (41.3) | 53 (34.2) | 16 (33.3) | 107 (55.6) | | 7 (33.8) | 1 (11.6) | 114 (37.4) | 8 (15.4) | 4 (15.7) |
| Overweight (25.0–29.9) | 7 (19.8) | 25 (22.5) | 23 (36.5) | 41 (29.5) | 60 (38.5) | 21 (31.6) | 45 (24.5) | | 5 (19.2) | 5 (53.7) | 109 (35.6) | 10 (18.7) | 3 (8.5) |
| Obese  (≥30) | 1 (1.6) | 18 (13.9) | 14 (19.6) | 23 (16.8) | 42 (24.4) | 43 (28.6) | 21 (9.1) | | 5 (35.6) | 7 (34.6) | 73 (22.5) | 19 (44.5) | 16(71.1) |

* The sample *n* is unweighted in this table. BMI: Body Mass Index.
